# Outcomes and costs in specialized burn care: Adapting the Quality Cost Indicator (QCI) model for burn care

**DOI:** 10.1371/journal.pone.0333660

**Published:** 2025-10-08

**Authors:** Raaba S. M. Thambithurai, Willem H. P. van Veghel, Denise van Uden, Jean-Bart Bügel, Anouk Pijpe, Marianne K. Nieuwenhuis, Cornelis H. van der Vlies, Margriet E. van Baar, Angelique E. A. M. Weel-Koenders, on behalf of the National Burn Care, Education & Research group, the Netherlands

**Affiliations:** 1 Alliance of Dutch Burn Care (ADBC), Burn Centre, Maasstad Hospital, Rotterdam, the Netherlands; 2 Erasmus School of Health Policy and Management, Erasmus University Rotterdam, Rotterdam, the Netherlands; 3 Department of Finance and Control, Franciscus Gasthuis en Vlietland Hospital, Rotterdam, the Netherlands; 4 Alliance of Dutch Burn Care (ADBC), Burn Centre, Red Cross Hospital, Beverwijk, the Netherlands; 5 Department of Plastic, Reconstructive and Hand Surgery, Amsterdam UMC location Vrije Universiteit Amsterdam, Amsterdam, the Netherlands; 6 Amsterdam Movement Sciences (AMS), Tissue Function and Regeneration, Amsterdam, the Netherlands; 7 Alliance of Dutch Burn Care (ADBC), Burn Centre, Martini Hospital, Groningen, The Netherlands; 8 Hanze University of Applied Sciences, Research Group Healthy Ageing, Allied Health Care and Nursing, Groningen, the Netherlands; 9 Department for Human Movement Sciences, University of Groningen, University Medical Center Groningen, Groningen, the Netherlands; 10 Departments of Trauma and Burn Surgery, Maasstad Hospital, Rotterdam, the Netherlands; 11 Trauma Research Unit Department of Surgery, Erasmus MC, University Medical Centre Rotterdam, Rotterdam, the Netherlands; 12 Department of Public Health, Erasmus MC, University Medical Centre Rotterdam, Rotterdam, the Netherlands; 13 Department of Rheumatology, Maasstad Hospital, Rotterdam, the Netherlands; University of California Davis, UNITED STATES OF AMERICA

## Abstract

The Quality Cost Indicator (QCI) model supports value-based health care at the institutional level, by calculating disease-specific health outcomes per unit cost over time. The aim of this study was to adapt the QCI model for specialized burn care (the BC-QCI model) and explore its utilization using real-world data. Burn care outcome indicators were selected through an iterative process with multiple stakeholders. Threshold values were established per outcome indicator and average total healthcare costs were calculated. A cohort of adult burn patients (n = 1449) admitted for at least one day and/or had undergone surgery in Dutch burn centers between 2020 and 2023 was used, with a follow-up period of 12 months. The proportion of patients who achieved textbook outcome (i.e., having achieved all the outcome indicators), the average total costs per patient, and QCI values were calculated. Of all patients, 54% achieved all five outcome indicators (i.e., length of stay, wound infections, other complications, discharge destination, and predicted mortality). The most successful outcome indicator was ‘predicted mortality’ (passed by 99% of the population), the least successful outcome indicator was ‘length of stay’ (62%). The patients who failed to achieve one or more outcome indicators (46%) had significantly higher average total costs compared to the patients who achieved textbook outcome (54%) (€50,134 [€47,810-€52,850] vs. €11,721 [€11,096-€12,429]). The BC-QCI model is a solid foundation to provide insights into the outcomes and costs for specialized burn care at the institutional level.

## Introduction

Burn injuries are a worldwide public health problem [1,2]. In many high-income countries, burn mortality rates have been decreasing as a result of improvements in burn treatments [3]. Patients with severe burns require complex, highly specialized care such as intensive care, surgical wound care, frequent labor-intensive wound dressing sessions and have a long rehabilitation period [4,5]. Patients with burn injuries are heterogeneous, due to variations in age, burn mechanism, extent and depth of the burn, while burns themselves are also heterogeneous and continuously subject to change [2,6,7].

Severe burn injuries are associated with high costs [5]. There is an increasing attention towards achieving sustainable healthcare costs, due to its increased complexity and expenses [5]. In the Netherlands, burn injuries can be treated in general practice, a general hospital or a specialized burn center. Dutch primary care is covered by the obligatory health insurance [8]. For secondary care, people pay the full cost up to the annual deductible (€385) [9]. Health insurance covers the costs of the acute burn phase and the majority of the aftercare phase [10]. To control the costs of burn care and to improve cost-effectiveness, more insights into the costs and outcomes are needed at the institutional level [5].

Health technology assessment (HTA) provides tools to evaluate healthcare interventions. In recent years, there has been an increase in these developments and the use of priority setting tools, such as cost-effectiveness analysis (CEA). However, there are limited practical tools that can be used at the institutional level (meso level) to set priorities. In various health conditions, research mostly focuses on macro (national) and micro (patient) levels [11,12].

A broad range of burn care quality indicators is available, encompassing the complete patient journey from the assessment of burns to the aftercare provided [13,14]. With these quality indicators the quality of care can be measured, and improvements of care can be stimulated. An optimal balance between these quality indicators and healthcare costs is essential to provide sustainable and accessible care, which embodies value-based health care (VBHC) [12,15]. VBHC strives to maximize patient-relevant care, per unit cost of the total care path [16]. VBHC measures relevant disease or care path-specific outcomes and healthcare utilization costs, making it practicable. Moreover, information at meso level is more easily obtained. This makes it possible to direct cost management strategies to achieve process and quality improvements [17].

Recently, the Quality Cost Indicator (QCI) model was developed, which is based on the principles of VBHC [18]. The QCI model is developed to pragmatically set priorities at the meso level. Furthermore, the model is applicable to a wide variety of care paths, due to the well-defined care pathways and measurements of disease specific clinical outcomes. Using the QCI model, the association between care path specific health outcomes and healthcare costs can be investigated. Insights into this association can help to support clinical and managerial decision making based on VBHC principles. It is also suitable for benchmarking between hospitals, as long as an uniform core outcome set is used.

So far, the QCI model is only used in breast cancer and bariatric surgery [18]. The aim of this study is to adapt the QCI model for burn care (Burn Care Quality Cost Indicator model, abbreviated as BC-QCI) and to explore its utility in specialized burn care using real-world data.

## Materials and methods

### Study design and population

This registry-based cohort study was conducted using real-world data from the Dutch Burn Repository R3 and the Burn Centers Outcomes Registry the Netherlands (BORN). The cohort consisted of adult burn patients (≥ 18 years) with acute burn injuries who were admitted for at least one day and/or had undergone surgery in one of the Dutch burn centers (Maasstad Hospital Rotterdam, Red Cross Hospital Beverwijk or Martini Hospital Groningen) between 1 January 2020 and 30 June 2023. The episode of care, as used in the BC-QCI model, started from the initiation of specialized burn care and ended at 12 months thereafter. In case of multiple burn care episodes, the first episode of care was considered. Patients missing data on one or more clinical outcome indicators were excluded from the analysis. The data was accessed for research purposes on June 6, 2024. The authors did not have access to any information that could identify individual participants during or after data collection. The Medical Research Ethics Committees United (MEC-U) judged that this study (number W23.252) did not fall under the scope of the Dutch Medical Research Involving Human subjects Act (WMO). The institutional review boards of the three participating hospitals approved this study.

### The QCI model

Central concepts in the QCI model include textbook outcome, average total costs, QCI date and QCI period. Textbook outcome (TO) is defined as achieving all the defined health outcome indicators. The average total costs is the sum of the total healthcare costs divided by total number of patients. The QCI date is a specific start date for a care path for a patient. The QCI period is the follow-up period in which the outcomes and costs should be determined [18].

The QCI value is calculated by the following ratio:


$$\text{QCI value }=\frac{proportion\;of\;textbook\;outcome\;*\;\text{1}\text{0}\text{0}}{Average\;total\;costs\;(per\;thousand\;Euros)}$$

### The adaptation of the QCI model into the BC-QCI model

The adaptation process of the BC-QCI model involved the following steps: the selection of health outcome indicators for burn care, the identification of their threshold values, and the determination of average total costs, QCI start date and QCI follow-up period.

1 **Selection of health outcome indicators for burn care**

To select burn care specific health outcome indicators, several steps were taken with multiple stakeholders. These steps are illustrated in S1 Appendix. First, the literature was reviewed to select candidate outcomes. Candidate outcomes were: ‘weight loss’, ‘length of stay’, ‘unplanned readmission’, ‘wound infection’ (as clinically diagnosed by burn care professional, based on a combination of clinical observations of infection and a positive wound swab), ‘proceed to palliative care’, ‘time until wound healing’, ‘complications’ (e.g., lung emboli and vanishing graft) ‘dermal preservation’, ‘unplanned reoperation’ and ‘number of wound dressing changes’ [14,19]. These candidate outcomes were discussed during two bi-annual meetings with the project team, which consisted of burn care professionals, burn survivors, healthcare managers, and burn care researchers. Next, five consecutive meetings were held separately with burn care professionals, burn survivors and healthcare managers, including a total of 17 participants. To assign equal value to all stakeholders, each group was asked to provide a top five of the most important health outcome indicators, from their perspective, that describe a successful burn treatment (i.e., quality of care). The total set of outcome indicators included those for which consensus was reached: ‘time until wound healing’, ‘length of stay’ (LOS), ‘complications’, ‘unplanned reoperation’, ‘wound infection’, ‘discharge destination’, ‘predicted mortality’ (determined by Revised Baux [20]), and ‘quality of life’. The patient reported outcome measure (PROM), the EuroQoL-5D-5L, was added to the set as an important outcome indicator of quality of life, as it can be utilized in cost-effectiveness analyses. However, some indicators had to be excluded from the set: ‘time until wound healing’ and ‘unplanned reoperation’ due to data unavailability and ‘quality of life’ due to incomplete data. The final set of included outcome indicators in this study as measures of quality of care were therefore ‘length of stay’, ‘wound infection’, ‘other complications’, ‘discharge destination’ and ‘predicted mortality’.

1. **Identification of threshold values**

Threshold values for each health outcome indicator were identified to determine if patients achieved the outcome indicator. To identify thresholds values for the outcome indicators, the literature was first reviewed for all outcome indicators. If there was no threshold value found in the literature, a candidate threshold value was established by consulting a burn physician. Subsequently, the candidate threshold values were discussed with the project team and burn care professionals to be finalized. An overview of the established health outcome indicator set, and the associated threshold values can be found in Table 1.

**Table 1:** The established health outcome indicator set and the associated threshold values.

Health outcome indicator burn care	Threshold value
Length of stay	If the actual LOS is longer than the predicted LOS, the treatment failed to achieve the clinical outcome indicator. Prediction is based on the model of Taylor et al. (2017) [21].
Wound infection	If a wound infection occurred during the QCI period due to the primary treatment, the treatment failed to achieve the clinical outcome indicator.
Other complications^a^	If complications occurred during the QCI period due to the primary treatment, the treatment failed to achieve the clinical outcome indicator.
Discharge destination	If a patient is discharged to a destination other than the usual residence, the primary treatment failed to achieve the clinical outcome indicator.
Predicted mortality	If a patient passed away during the QCI period due to the primary treatment or complications following the primary treatment or while the predicted risk of mortality was ≤ 25%, the treatment failed to achieve the clinical outcome indicator. Prediction is based on Revised Baux model 2010 [20,22].

^a^ *Other complications included Pressure ulcer staging, Acute Respiratory Distress Syndrome’ (not a complication if ≥20% TBSA), Vanishing graft, Wound enlargement, Psychotic behavior/delirium, Polyneuropathy (not a complication if ≥20% TBSA), Other neurological injury, Bleeding/ Ulcer, Paralytic ileus, Pressure ulcer, Urinary tract infection, Other tract, Other urogenital, Partial graft take, Diabetes after trauma, Other endocrine, Pneumothorax, Pulmonary embolism, Other pulmonary, Thrombosis, Embolism, Acute renal failure, Ischemia, Arrhythmia, Other circulatory, Bronchopneumonia, Aspiration pneumonia, Other complications. Acute Respiratory Distress Syndrome and Polyneuropathy were not classified as complication in patients with TBSA ≥20%, as these occurred frequently in this subgroup.*

1. **The determination of average total costs, QCI start date, and QCI follow-up period**

The cost analysis was performed from a health care perspective. The total costs of specialized burn care were calculated for each patient, which included direct medical costs (burn centre stay, treatment, clinical consultation and outpatient burn care). Burn care costs were calculated by multiplying the volumes of healthcare used with the corresponding unit prices. The unit prices were derived from the Dutch guidelines and previous studies [5,23]. The unit prices of previous studies were updated by inflation correction to 2023. Subsequently, the average total costs were calculated. The BC-QCI date is defined as the start date of the first care episode. The BC-QCI period is 12 months after initiation of specialized burn care.

### Data collection

Data on patient and burn characteristics, as well as the clinical outcome indicators were collected from the Dutch Burn Repository R3 (DBR R3).

### Data analysis

Data were presented per year and per quarter to monitor outcomes over time.

#### Case-mix adjustments.

Case-mix adjustments were done to increase the comparability of outcomes and costs over time. The following variables were used for the case-mix adjustment [23].

- Age (18–60 years, > 60 years)
- %TBSA (0–5%, 6–10%, 11–20%, > 20%)
- Etiology (Flame, others)

#### Categories of outcome indicators.

To compare patients per outcome indicator, a minimum of five patients per outcome category (combining both achieved and failed to achieve) was required. To meet this requirement, some outcome indicators were combined, creating a new category, ‘other’, which consisted of patients from the outcome indicators ‘complications,’ ‘wound infection,’ and ‘predicted mortality’.

#### Statistical testing.

All variables were tested for normality by using the Kolmogrov-Smirnov test. Normally distributed continuous data were reported as mean and standard deviation (SD). Skewed continuous data were described as median and 25^th^ −75^th^ percentiles. Categorical data were described as numbers and percentages. To analyze if costs differed significantly between patients who achieved vs. patients who failed to achieve the outcome indicators, the Kruskal-Wallis analysis was used, with an alpha value of 0.05 (two sided). In addition, bootstrapping (1000 times) was performed to calculate the 95% confidence interval (CI) for costs. Data analysis was performed using SPSS Statistics version 29 (IBM).

## Results

### Patient population

The dataset contained a total of 1494 adult patients who were admitted in the three Dutch burn centers between January 1, 2020, and June 30, 2023. After the exclusion of 45 patients (3%) with missing data on ‘length of stay’, ‘complications’, ‘wound infection’, ‘discharge destination’, or ‘predicted mortality’, 1449 patients were eventually included in this study. Their median age was 48 years (IQR 31–63 years), 64% of the patients were male, the median %TBSA was 4% (IQR 1%−8%) and the median LOS was 4 days (IQR 0–17 days). Of all the patients, 1028 (71%) patients received one or more surgical interventions (i.e., escharotomy, excision, skin grafting, reoperation). Seventeen percent of all patients were admitted to the intensive care unit (ICU) (Table 2).

**Table 2:** Patient and injury characteristics (n = 1449).

Gender	
Male, n(%)	931 (64%)
Age	
Median age at injury (IQR)	48 (31–63) years
Age categorized, n(%)	
18-60 years	1033 (71%)
>60 years	416 (29%)
Etiology	
Fire/Flame	669 (46%)
Other^a^	780 (54%)
%TBSA	
Median %TBSA (IQR)	4% (1%−8%)
%TBSA categorized n(%)	
0-5%	923 (64%)
6-10%	231 (16%)
11-20%	180 (12%)
>20%	115 (8%)
Inhalation injury (clinically diagnosed) n(%)	63 (4%)
Median length of hospital stay (IQR)	4 (0–17) days
Median length of ICU stay (n = 252) (IQR)	4 (2–13) days
Nº of surgeries per patient (n = 1028), n(%)	
1	760 (74%)
2	132 (13%)
>2	136 (13%)
Complication n(%)	196 (14%)
Wound infection (clinically diagnosed) n(%)	31 (2%)
Mortality n(%)	28 (2%)

^a^Consists of scald, fat/hot oil, contact and chemical and electrical burns

### Health outcome indicators over time

Of all patients, 54% successfully passed all outcome indicators, resulting in a textbook outcome (Table 3). The proportion of textbook outcome ranged between 44%−66% over time. Moreover, almost all outcome indicators were achieved by the majority of patients. The most successful indicator was predicted mortality, with a mean of 99% (range 97%−100%) of the patients passing this outcome. The least successful indicator was length of stay, with a mean of 62% (range 53%−74%) of the patients passing this outcome.

**Table 3:** Health outcome indicators Q1 2020 – Q2 2023 (n = 1449).

Number (achieved/failed to achieve) and crude percentage of patients achieving the respective health outcome indicator						
Outcome indicator		Q1	Q2	Q3	Q4	Total
Length of stay	2020	55/49	62/46	65/44	54/38	**892/557** **62%**
		53%	57%	60%	59%	
	2021	68/24	63/39	40/34	59/37	
		74%	62%	54%	62%	
	2022	81/38	77/45	66/46	50/39	
		68%	63%	59%	56%	
	2023	73/36	79/42	–	–	
		67%	65%			
Wound infection	2020	103/1	107/1	105/4	88/4	**1418/31** **98%**
		99%	99%	96%	96%	
	2021	90/2	98/4	72/2	95/1	
		98%	96%	97%	99%	
	2022	117/2	119/3	111/1	87/2	
		98%	98%	99%	98%	
	2023	108/1	118/3	–	–	
		99%	98%			
Other complications	2020	92/12	94/14	98/11	83/9	**1292/157** **89%**
		89%	87%	90%	90%	
	2021	85/7	91/11	65/9	84/12	
		92%	89%	88%	88%	
	2022	106/13	115/7	103/9	78/11	
		89%	94%	92%	88%	
	2023	94/15	104/17	–	–	
		86%	86%			
Discharge destination	2020	100/4	101/7	104/5	84/8	**1367/82** **94%**
		96%	94%	96%	91%	
	2021	86/6	92/10	65/9	94/2	
		94%	90%	88%	98%	
	2022	112/7	118/4	106/6	85/4	
		94%	97%	95%	96%	
	2023	104/5	116/5	–	–	
		95%	96%			
Predicted mortality	2020	103/1	107/1	107/2	91/1	**1434/15** **99%**
		99%	99%	98%	99%	
	2021	89/3	102/0	74/0	94/2	
		97%	100%	100%	98%	
	2022	117/2	122/0	112/0	89/0	
		98%	100%	100%	100%	
	2023	106/3	121/0	–	–	
		97%	100%			
Proportion of textbook outcome	**2020**	**46/58**	**58/50**	**55/54**	**47/45**	**786/663** **54%**
		**44%**	**54%**	**51%**	**51%**	
	**2021**	**61/31**	**53/49**	**33/41**	**51/45**	
		**66%**	**52%**	**45%**	**53%**	
	**2022**	**72/47**	**72/50**	**62/50**	**46/43**	
		**61%**	**59%**	**55%**	**52%**	
	**2023**	**60/49**	**70/51**	**–**	**–**	
		**55%**	**58%**			

### Costs in burn care

The average total specialized burn care costs per patient were €29,297 [€ 28,007-€30,748] (Table 4). The major cost components were burn center stay (76% of the total average costs per patient) and burn treatment costs (21% of the total average costs), mainly due to surgical treatments. The unit costs per day was €3894 (ICU day) and €1245 (non-ICU day).

**Table 4:** Average costs per patient of specialized burn care (€, 2023).

Cost category	Total costs (€) (N = 1449)
Burn center stay	
ICU burn center days^a^	€7715
Non ICU burn center days^a^	€14,263
Readmittance days^b^	€15
Day care	€137
*Total burn center stay [95% CI]*	*€22,129 [€ 20,997- € 23,413]*
Treatment	
Diagnostic procedures	€964
Wound care	€1339
Surgical treatment	€3008
Blood products	€278
Pressure garments	€516
Silicon therapy	€144
Splints	€6
*Total treatment [95% CI]*	*€6255 [€6120- €6393]*
Clinical burn consultations	
*Total clinical consultation*	*€487*
Outpatient burn care	
Outpatient wound care	€266
Outpatient scar care	€84
After care nurse	€21
Plastic surgeon	€8
Other outpatient burn care^c^	€47
*Total outpatient burn care*	*€426*
*Total specialized burn care*	
Total (average) costs specialized burn care per patient *[95% CI]*	*€29,297 [€ 28,007* *- €30,748]*
	

^a^ *The costs of non ICU and ICU burn center stay consisted of personnel (including burn physicians), material (excluding wound care), equipment, food, laundry and medication costs per days including a 41.9% increase for housing and overhead*

^b^ *Readmittance days refer to hospital days of patients readmitted after discharge. The total readmittance costs were allocated across the entire study population, resulting in an average cost of €15 per patient.*

^c^ *Other outpatient burn care consists of psychologist, occupational therapist, physiotherapist, rehabilitation physician, skin therapist, social worker and dietitian.*

Next, the costs were determined per outcome category (combining both achieved and failed to achieve the defined outcome indicator) to provide insight into which subgroups of patients incurred the highest costs on average (S2 Appendix). For each outcome category, the group who achieved the defined outcome category had a lower average total cost compared to the patients who failed to achieve the defined outcome category. The outcome category with the highest incurred average total costs (€77,402 [€69,396-€85,170]) were patients who failed to achieve ‘discharge destination’. The average total costs per outcome category differed significantly within the subgroups.

**Fig 1:**
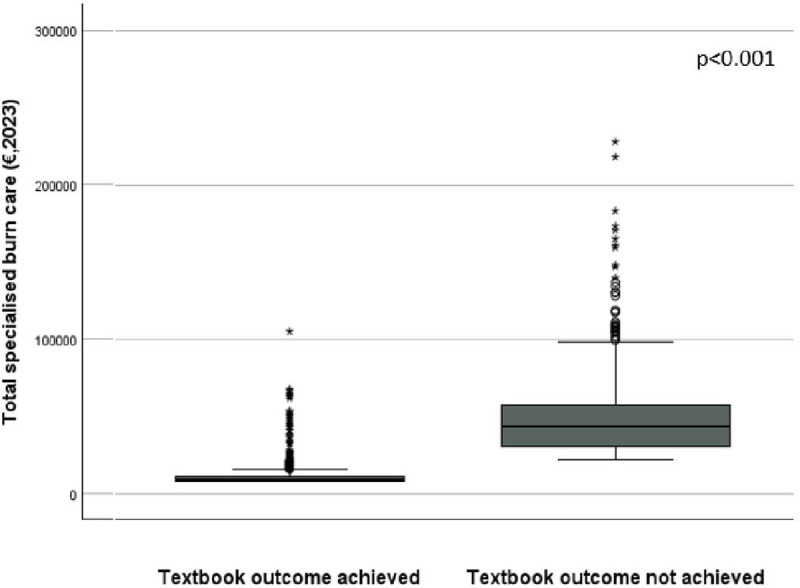
The average total costs of the group that achieved textbook outcome compared to the group that not achieved textbook outcome. The average total costs for the achieved textbook outcome group are €11,721, 95% CI [€ 11,096-€ 12,429]. The average total costs for the not achieved textbook outcome group are €50,134, 95% CI [€ 47,810-€52,850].

The group who achieved all outcome indicators, so achieved textbook outcome, had significantly lower costs on average compared to the group who failed to achieve one or more outcome indicators (€11,721 [€ 11,096-€ 12,429] vs. €50,134 [€ 47,810-€52,850]). Fig 1 illustrates that the group who achieved textbook outcome had less variations in costs compared to the group that failed to achieve textbook outcome. The group failing to achieve one or more health outcome indicators had on average a higher %TBSA, more flame accidents, more complications, more wound infections, more surgical treatment, a longer length of hospital stay and a longer ICU stay than the successful group (S3 Appendix).

### QCI values over time

**Fig 2:**
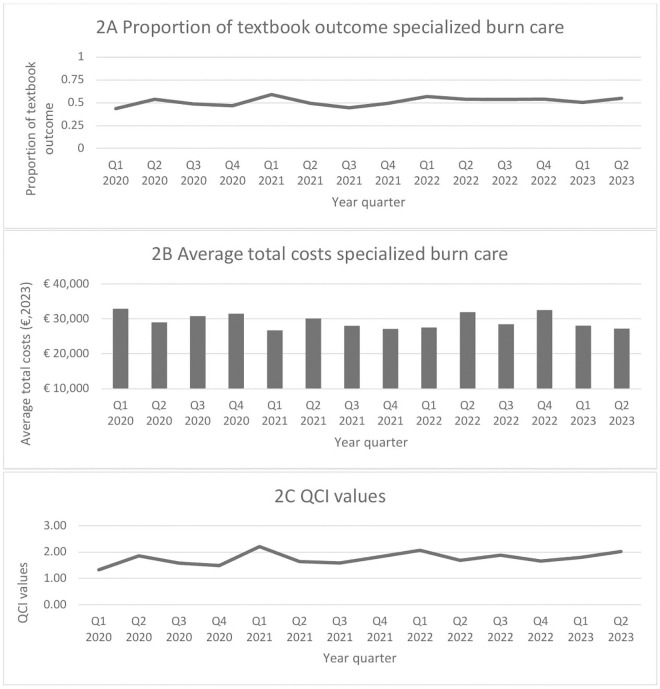
The proportion of textbook outcome, average total costs and obtained QCI values in specialized burn care per quarter. Panel 2A reflects the ratio of the number of patients who achieved textbook outcome to the total number of patients in that quarter. This ratio ranges between 0-1. The average total costs are illustrated in panel 2B. The ratio of the average total costs to the total number of patients per quarter are displayed. The costs are presented in euros. The QCI values in panel 2C are the ratio of the achieved textbook outcome to the average total costs (per thousand euros) per quarter.

QCI value is defined as the combination of the health outcome indicators relative to the cost, in order to gain insight in the value achieved per unit of cost spent. QCI values were calculated using the proportion of textbook outcome (Table 3) and the average total cost incurred by a specific population within a particular quartile. Within the total burn population there was minimal variation when it comes to achieving textbook outcome over time (Fig 2A). The lowest rate of textbook outcome is found in 2020 Q1, while the highest rate of textbook outcome appears in 2021 Q1, and in contrast, the lowest average costs were observed in 2021 Q1 (€26,696), while the highest average costs were found in 2020 Q1 (€32,848). The variation in obtained QCI values per quarter is presented in Fig 2, panel 2C. The QCI values seem to demonstrate greater variation from 2020 Q1 to 2022 Q1 compared to the period from Q2 2022–2023 Q2, which is likely due to costs. The lowest obtained QCI value was in 2020 Q1 and the highest level in 2021 Q1.

## Discussion

The QCI model was adapted to the BC-QCI model to explore its utility in specialized burn care using real-world data. Fifty-four percent of all patients passed all health outcome indicators. The most successful outcome indicator was ‘predicted mortality’ and the least successful outcome indicator was ‘length of stay’. The highest incurred costs in specialized burn care were related to burn center stay (76% of the total average costs per patient). In addition, the costs analysis per outcome category, adjusted for case-mix, showed that patients who failed to achieve textbook outcome, on average, had significantly higher total costs compared to patients achieving textbook outcome. Moreover, the cost of burn care accounted for the largest variation per QCI-value quartile.

The focus on VBHC is growing in healthcare, specifically in the Netherlands, but is mainly focused on outcome implementation and benchmarking [24]. Moreover, its implementation in hospital settings remains limited. In addition, VBHC and thus the patient perspective, represented by the use of PROMs, has also not yet been systematically applied in burn care. Priority setting tools, like the BC-QCI model, can be helpful to monitor the effects of VBHC initiatives. Moreover, the QCI model focuses on the association between outcome indicators and healthcare costs, which is often lacking in VBHC initiatives. Also, to further support VBHC in practice, Dutch burn care recently developed a VBHC burns core set, which consist for a large part of patient reported outcomes [15]. Although the use of PROMS is increasing, it is not yet widely available. Therefore, in the BC-QCI model, internationally recognized indicators were selected to enhance its generalizability in burn care [25].

The findings of this study showed that prolonged LOS resulted in ‘length of stay’ being the least successful outcome indicator. Currently, mortality rates have decreased due to improvements in burn treatments, and the focus has shifted to other quality indicators, such as LOS [3,26]. Our study showed that mortality indeed was low, as ‘predicted mortality’ was the most successful outcome indicator in the model. A recent scoping review reported that LOS is the most cited outcome indicator for measuring the quality of hospital care in acute burn care [27]. However, prolonged LOS remains common in burn care [28]. The most common prediction of LOS, i.e., 1 day of hospitalization per 1%TBSA, is often an underestimation [21]. Several studies have identified numerous factors that affect the LOS, such as age, sex, %TBSA, infections, comorbidities, depth of burn, and surgical procedures [29–32]. The influence of factors such as infections or comorbidities on LOS also resonates in the BC-QCI model, as these factors define textbook outcome. In this study, the threshold value for LOS was determined using a predictive model based on variables age, %TBSA, and inhalation injury. This LOS prediction model is based on data from the American Burn Association [21]. Dutch burn care is known for its conservative approach regarding grafting, as are several other European burn centers [33]. The selection of this predictive model, based on American data, therefore may not be ideal to apply on Dutch burn care and may have affected the outcomes. The LOS prediction model includes more variables than just %TBSA, yet several variables that affect LOS are not included in this LOS model. Therefore, further exploration with this LOS prediction model in our population is needed to determine its suitability.

In addition, when it comes to costs, we reported that one of the greatest cost drivers in burn care is LOS. This is also found in other studies [5,23,28]. To steer on costs, it is important to identify key cost drivers, as well as improvement initiatives focused on addressing them. Apart from LOS, other significant components of costs are operative procedures and wound care, which were also identified in the literature [34]. Besides steering on reducing the main cost components, the quality of care according to patients and clinicians should not be compromised. The BC-QCI could provide insights in this balance and helps steering towards VBHC. This way the model can be used beyond simply clarifying processes. Initiatives focusing on reducing prolonged length of stay and providing early surgical care while improving patient-related outcomes may be crucial for lowering burn care costs while improving care [50, 51]. Additionally, this study showed that the group that failed to achieve one or more outcomes had on average higher costs. Previous research on burn injury costs showed that %TBSA is an important determinant of costs [34]. Increases in %TBSA were associated with increases in costs [23]. Moreover, costs due to flame accidents were higher than other type of burns [23,35]. Age also significantly influences outcomes. Age-related comorbidities may impact burn patient outcomes (e.g., LOS increases with the number of comorbidities) [36,37]. This all could also explain the great variation in costs and its variation over time due to the heterogeneous nature of the burn population.

This study has several limitations that should be noted. First, candidate health outcome indictors were selected by reviewing the literature. Conducting a systematic review would have been more comprehensive. However, this was beyond the scope of this study. Furthermore, ‘unplanned reoperation’ and ‘time until wound healing’ were excluded due to unavailability of data. However, stakeholders indicated these outcomes were important indicators to measure outcomes. Therefore, it is important to measure these outcomes so that they can be included in future analyses. Moreover, ‘quality of life’ was excluded due to incomplete data, whereas the BC-QCI model is based on VBHC principles and therefore the patient’s perception should be taken into account. Second, it is important to be aware that the choice for alternative threshold values can lead to different results. This study is the first to apply the LOS prediction model to our population. Further exploration of this prediction model is needed in this setting. Third, we did not account for co-morbidity. Therefore, correction for case-mix is necessary to optimally compare groups of patients. Moreover, case-mix adjustments were limited to the variables age, %TBSA and etiology, also because of limitations in our sample size. However, other key clinical variables, such as burn depth, could be included in further research to allow for broader adjustments. Furthermore, in the BC-QCI model, both non-ICU patients and ICU patients are combined. A separate analysis was not performed, which could have provided additional insights into outcomes and costs for each group. We recommend performing separate analysis for ICU patients and non-ICU patients, allowing for a better understanding of the variations in cost patterns and clinical results between the two patient groups.

This study also has several strengths. A strength of this study is that a broad range of stakeholders participated in the development of the BC-QCI model. This model includes perspectives of patients, managers and care professionals in determining the relevant outcome set of their care path, which is based on VBHC principles. Moreover, the adaptation of the QCI model to the BC-QCI model and its utilization in specialized burn care results in a practical tool for steering outcomes in relation to costs at the meso level. Also, by using real world data, the findings showed that this framework is applicable for acute care. Finally, this study contributed to filling the gap in health technology assessment models about practical tools to steer on outcomes and costs at the meso level.

### Conclusion

The utilization of the BC-QCI model showed that a solid foundation has been established in specialized burn care to monitor and steer costs and outcomes from a VBHC perspective. The joint analysis of costs and outcomes provides insights into the value achieved per unit of cost spent at the meso level. This model can be used in future applications to assess healthcare interventions based on VBHC principles. To promote its adoption in hospitals, this model can be integrated into hospital dashboards to monitor and improve care. Associations between outcomes and costs can help to support clinical and managerial decision making in specialized burn care.

## Supporting information

S1 Appendix Flowchart of the process for determining health outcome indicators in burn care. (DOCX)

S2 Appendix Cost per outcome category. (DOCX)

S3 Appendix Patient and injury characteristics textbook outcome population. (DOCX)
