# Supplementary material for: Outcomes and costs in specialized burn care: Adapting the Quality Cost Indicator (QCI) model for burn care
Source: PLoS One. 2025 Oct 8;20(10):e0333660. doi: 10.1371/journal.pone.0333660 (PMC12507314; doi:10.1371/journal.pone.0333660)
Supplement: S1 Appendix — (DOCX) [file pone.0333660.s001.docx]

**S1 Appendix. Flowchart of the process for determining health outcome indicators in burn care**

^1.The possible indicators were: weight loss, length of stay, unplanned readmission, wound infection, proceed to palliative care, time until wound healing, complications, dermal preservation, unplanned reoperation and number of wound dressing treatments (^[^14^](#_ENREF_14)^,^ [^19-21^](#_ENREF_19)^).^

^2.^ ^The list of ten candidate indicators was: unplanned reoperation, unplanned readmission, length of stay, wound infection, complications, dermal preservation, number of wound dressing treatments, time until wound healing, weight loss and proceed to palliative care (^[^14^](#_ENREF_14)^,^ [^19-21^](#_ENREF_19)^).^

^3. The predefined set consisted of: unplanned reoperation, unplanned readmission, length of stay, wound infection, complications, graft take, number of wound dressing treatments, time until wound healing, weight loss and proceed to palliative care.^

^4. The seven indicators included: time until wound healing, admission period, complications, unplanned reoperation, wound infection, discharge destination and predicted mortality.^

^5. The final set: time until wound healing, admission period, complications, unplanned reoperation, wound infection, discharge destination, predicted mortality and quality of life.^
