# Supplementary material for: Outcomes and costs in specialized burn care: Adapting the Quality Cost Indicator (QCI) model for burn care
Source: PLoS One. 2025 Oct 8;20(10):e0333660. doi: 10.1371/journal.pone.0333660 (PMC12507314; doi:10.1371/journal.pone.0333660)
Supplement: S2 Appendix — (DOCX) [file pone.0333660.s002.docx]

**S2 Appendix. Cost per outcome category**

| **Cost per outcome category** | | | | | | | | |
| --- | --- | --- | --- | --- | --- | --- | --- | --- |
| **Population** | | **Outcome category** | **Number of patients** | **Average Costs** | **95% CI^a^** | **Standard Deviation Costs** | **Range of Costs** | **P Value** |
| Admission period | | Failed to achieve | 557 | €53,968 | [€51,651- €56.642] | €30,504 | €325,247 | <0.001 |
|  |  | Achieved | 892 | €13,558 | [€12,863- €14,339] | €11,174 | €156,967 |  |
|  |  | | | | | | | |
| Discharge destination | | Failed to achieve | 82 | €77,402 | [€69,396 - €85,170] | €41,218 | €203,207 | <0.001 |
|  |  | Achieved | 1367 | €26,412 | [€25,278 - €27,544] | €23,158 | €326,032 |  |
|  |  | | | | | | | |
| Other | | Failed to achieve | 182 | €51,524 | [€46,915 - €56,009] | €38,193 | €326,032 | <0.001 |
|  |  | Achieved | 1267 | €27,221 | [€26,112 - €28,347] | €21,598 | €167,082 |  |
|  |  | | | | | | | |
| Textbook outcome | | Failed to achieve | 663 | €50,134 | [€47,810 - €52,850] | €30,214 | €326,032 | <0.001 |
|  |  | Achieved | 786 | €11,721 | [€11,096 - €12,429] | €9406 | €100,612 |  |

^a^ Bootstrapping (1000 times) was performed to calculate the 95% confidence interval (CI) for costs
