# Supplementary material for: Outcomes and costs in specialized burn care: Adapting the Quality Cost Indicator (QCI) model for burn care
Source: PLoS One. 2025 Oct 8;20(10):e0333660. doi: 10.1371/journal.pone.0333660 (PMC12507314; doi:10.1371/journal.pone.0333660)
Supplement: S3 Appendix — (DOCX) [file pone.0333660.s003.docx]

**S3 Appendix. Patient and injury characteristics textbook outcome population**

| Patient and injury characteristics textbook outcome population | | | |
| --- | --- | --- | --- |
|  | **Textbook outcome**  **achieved**  **(n=786)** | **Textbook outcome not achieved**  **(n=663)** |  |
| Gender |  |  |  |
| Male, n(%) | 499 (64%) | 432 (65%) |  |
| Age |  |  |  |
| Median age at injury (SD) | 46 (29-61) years | 50 (33-68) years |  |
| Age categorized, n(%) |  |  |  |
| 18-60 years | 581 (74%) | 452 (68%) |  |
| >60 years | 205 (26%) | 211 (32%) |  |
| Etiology |  |  |  |
| Fire/Flame | 321 (41%) | 348 (53%) |  |
| Other* | 465 (59%) | 315 (48%) |  |
| %TBSA |  |  |  |
| Median %TBSA (IQR) | 2% (1%-5%) | 6% (2-12%) |  |
| %TBSA categorized n(%) |  |  |  |
| 0-5% | 600 (76%) | 323 (49%) |  |
| >5-10% | 94 (12%) | 137 (21%) |  |
| >10-20% | 53 (7%) | 27 (19%) |  |
| >20% | 39 (5%) | 76 (12%) |  |
|  |  |  |  |
| Inhalation injury n(%) | 24 (3%) | 39 (6%) |  |
|  |  |  |  |
| Median length of hospital stay (IQR) | 0 (0-3) days | 17 (7-28) days |  |
|  |  |  |  |
| Median length of ICU stay (IQR) | 3 (2-5) days | 6 (3-23) days |  |
|  |  |  |  |
| Nº of surgeries per patient, n(%) | n=536 | n=492 (74%) |  |
| 1 | 452 (84%) | 308 (63%) |  |
| 2 | 50 (9%) | 82 (17%) |  |
| >2 | 32 (6%) | 102 (21%) |  |
|  |  |  |  |
| Complication n(%) | 11 (1%) | 185 (28%) |  |
|  |  |  |  |
| Wound infection n(%) | 0 (0%) | 31 (5%) |  |
|  |  |  |  |
| Mortality n(%) | 9 (1%) | 19 (3%) |  |

* Consists of scald, fat/hot oil, contact and chemical and electrical burns
